# Chromosome 15q13 microduplication in a fetus with cardiac rhabdomyoma: a case report

**DOI:** 10.1186/s13039-019-0437-1

**Published:** 2019-05-27

**Authors:** Chen-Zhao Lin, Bi-Ru Qi, Jian-Su Hu, Yu-Dian Huang, Xiu-Qiong Huang

**Affiliations:** 1 Department of Obstetrics and Gynecology, Affiliated Fuzhou First Hospital of Fujian Medical University, No. 190 Dadao Road, Taijiang District, Fuzhou, Fujian Province 350009 People’s Republic of China; 2 Department of Ultrasound, Affiliated Fuzhou First Hospital of Fujian Medical University, Fuzhou, 350009 Fujian Province People’s Republic of China; 3 Department of Pathology, Affiliated Fuzhou First Hospital of Fujian Medical University, Fuzhou, 350009 Fujian Province People’s Republic of China; 4 Department of Laboratory Medicine, Affiliated Fuzhou First Hospital of Fujian Medical University, Fuzhou, 350009 Fujian Province People’s Republic of China

**Keywords:** Chromosome 15q13 microduplication, Cardiac rhabdomyoma, Copy number variation, Chromosomal microarray analysis

## Abstract

**Background:**

Copy number variation (CNV) is a complex genomic rearrangement that has been linked to a large number of human diseases. Chromosome 15q13 microduplication is a rare form of CNV, which has been proved to be associated with multiple human disorders; however, the association between chromosome 15q13 microduplication and cardiac disorders has not been fully understood.

**Case presentation:**

A fetus with fetal cardiac developmental defects was detected by Color Doppler ultrasound imaging; however, further chromosomal G-banding revealed no abnormal karyotype. Then, chromosomal microarray analysis (CMA) was performed and revealed a 1.8 Mb-duplication of the chromosome 15q13.2q13.3 region containing 7 genes (*TRPM1*, *KLF13*, *OTUD7A*, *CHRNA7*, *FAN1*, *MIR211* and *RAHGAP11A*). Cardiac ultrasound follow-up displayed significant enlargement of the space-occupying lesion in the fetal heart with extension of the gestational age, and the space-occupying lesion was finally pathologically diagnosed as cardiac rhabdomyoma. Next-generation sequencing revealed no mutations in the *TSC1* or *TSC2* gene in the fetus, the mother or the father.

**Conclusions:**

This is the first report to demonstrate the potential association between chromosome 15q13 microduplication and fetal cardiac rhabdomyoma. It is recommended that CMA be employed in fetuses with abnormal cardiac development diagnosed by routine cardiac color Doppler ultrasound imaging for early detection of congenital genetic abnormality, which may provide valuable information for prenatal diagnostic consultation and the decision on pregnancy termination.

## Background

Copy number variation (CNV), a type of genomic structural variation, has been defined as a DNA segment of one kilobase (kb) or larger that is present at a variable copy number as compared to a reference genome [1]. As a complex genomic rearrangement [2], CNV has been linked to a large number of human diseases [3–5]. Chromosome 15q13 microduplication, a rare form of CNV, has been associated with cognitive impairment, autism, seizures, and attention-deficit hyperactivity disorder, and oral clefts [6–14], and the common recurrent BP4-BP5 duplication in the chromosomal region 15q13.3 was detected in a 14-year-old male with Tetralogy of Fallot and developmental delay [15]. However, the association between chromosome 15q13 microduplication and cardiac disorders has not been fully understood. Here, we report a fetus with cardiac rhabdomyoma who had a 1.8 Mb-duplication of the chromosome 15q13.2q13.3 region as detected by chromosomal microarray analysis (CMA).

## Case presentation

**Fig. 1:**
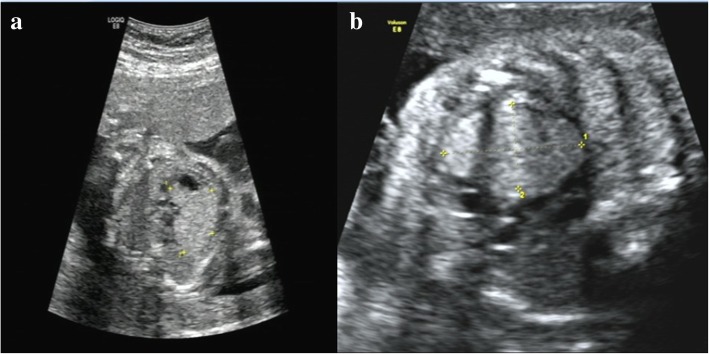
Color Doppler ultrasound imaging. **a** on November 3, 2017 (23 + 4 weeks of gestation), color Doppler ultrasound imaging showed a hyperechogenic mass in the fetal left ventricle, measuring 1.8 cm × 1.57 cm, broadening of the left lateral ventricle (1.11 cm) and a strong dot-like echo in the left ventricle; **b** on December 7, 2017, color Doppler ultrasound reexaminations displayed multiple strong echoes in the fetal left ventricle (measuring 3.3 cm × 2.03 cm), compression of the left ventricular outflow tract, obvious enlargement of the tumor, and a 0.92 cm internal diameter of posterior horn of the left lateral ventricle

**Fig. 2:**
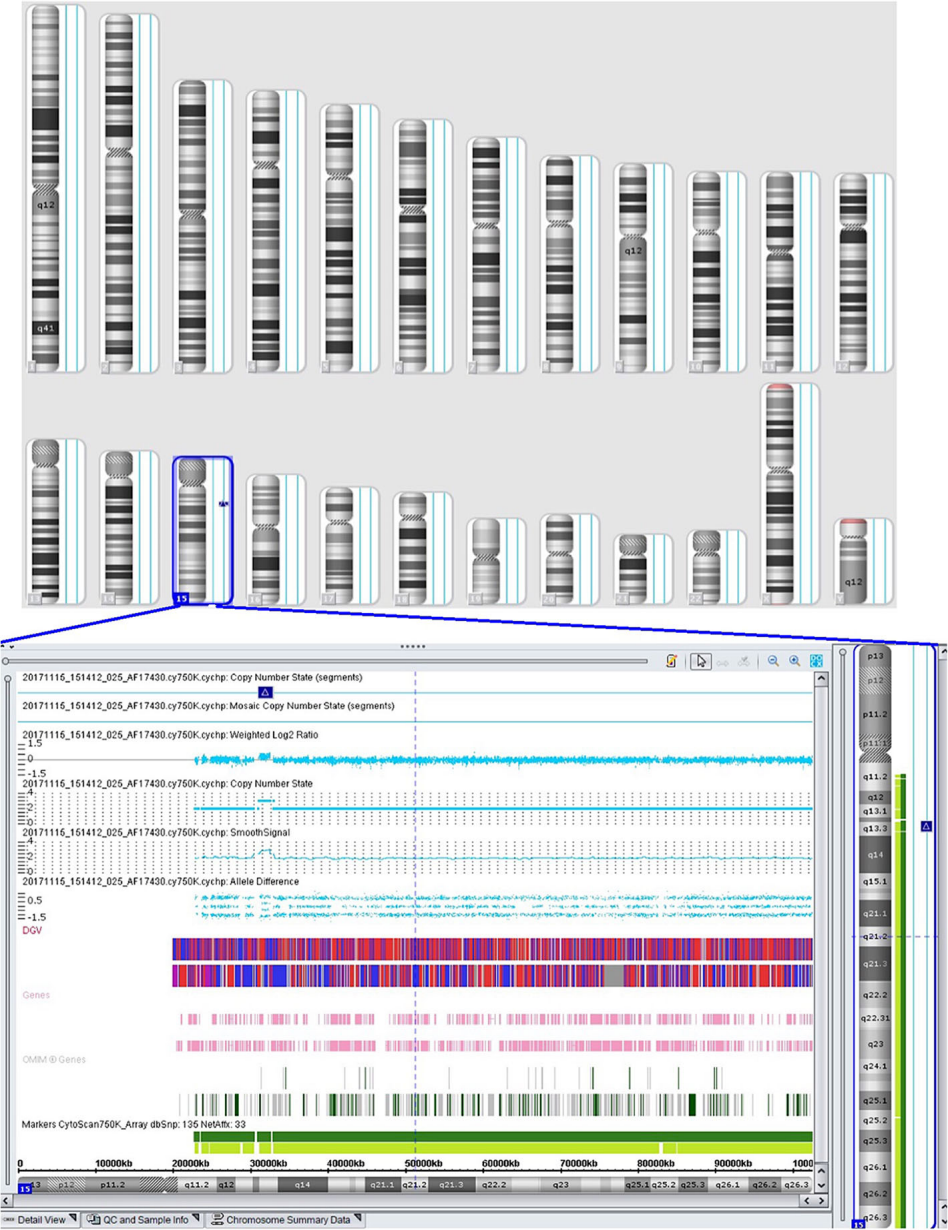
Chromosomal microarray analysis detects a 1.8 Mb-duplication of the chromosome 15q13.2q13.3 region containing 7 genes, which occurs in the region between BP4-BP5 on chromosome 15q13.3

A 33-year-old pregnant woman, G_1_P_0_, at a gestational age of 23 + 4 weeks was referred to our hospital on November 3, 2017. Color Doppler ultrasound imaging showed a hyperechogenic mass in the fetal left ventricle, measuring 1.8 cm × 1.57 cm, broadening of the left lateral ventricle (1.11 cm) and a strong dot-like echo in the left ventricle (Fig. 1a), and cardiac rhabdomyoma was suspected. On November 7, 2017, approximately 30 mL of amniotic fluid was collected by ultrasound-guided transabdominal puncture for conventional chromosomal G-banding karyotype analysis and CMA with the BioChip Detection System of Affymetrix GeneChip following the manufacturer’s instructions (Zhejiang Biosan Biochemical Technologies Co., Ltd.; Hangzhou, China). G-banding analysis revealed a 46, XY karyotype, with no abnormal karyotype detected, and CMA detected a 1.8 Mb-duplication of the chromosome 15q13.2q13.3 region (arr [hg19] 15q13.2q13.3(31,073,668-32,920,694)× 3) containing 7 genes (*TRPM1*, *KLF13*, *OTUD7A*, *CHRNA7*, *FAN1*, *MIR211* and *RAHGAP11A*), which occurred in the region between BP4-BP5 on chromosome 15q13.3 (Fig. 2).

**Fig. 3:**
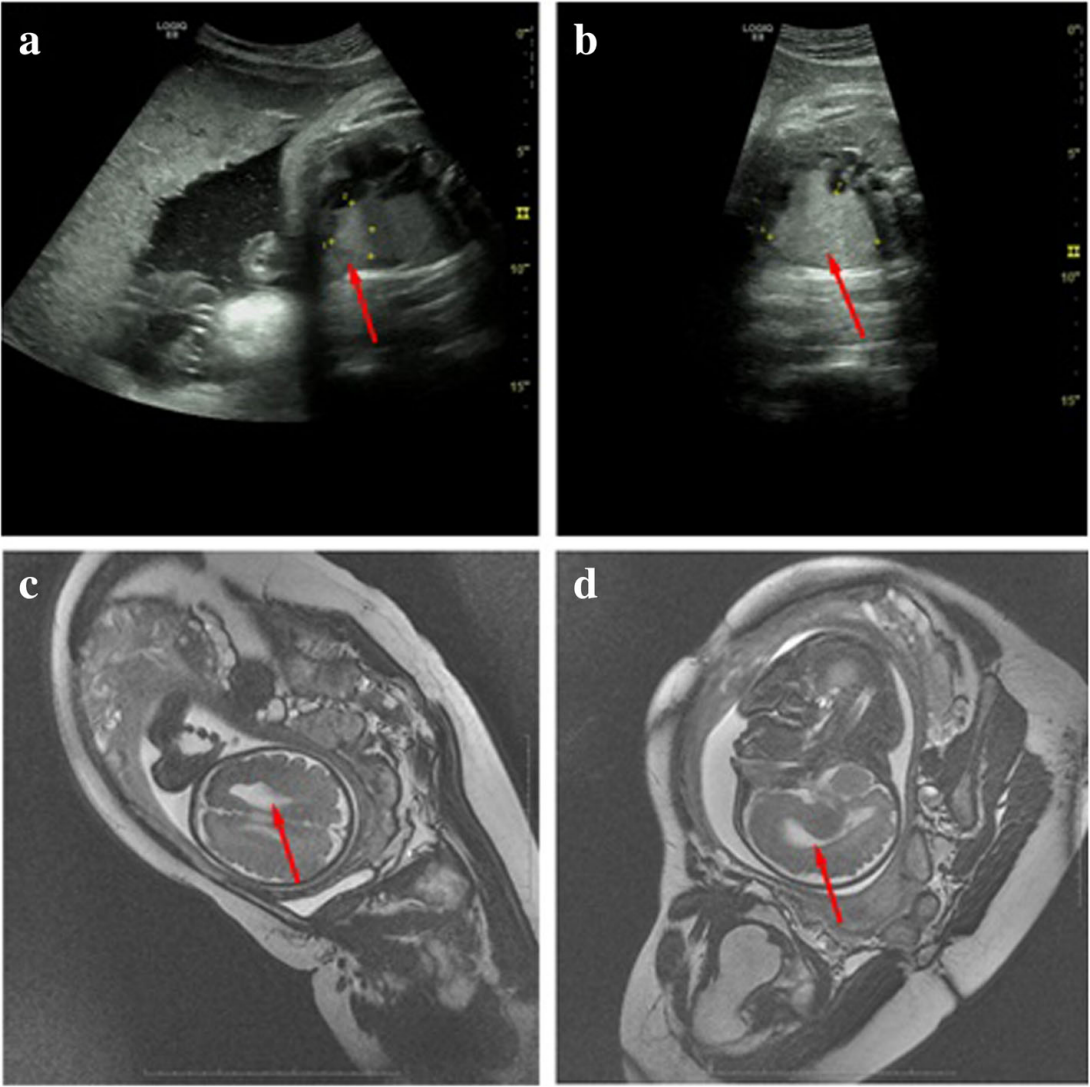
Color Doppler ultrasound and fetal brain magnetic resonance imaging examinations. **a** and **b** On January 25, 2018 (35 + 2 weeks of gestation), color Doppler ultrasound reexaminations of the fetal heart display multiple hyperechogenic masses (measuring 4.35 cm × 3.13 cm) in the fetal left ventricle, abnormal morphology of anterior horn of the left lateral ventricle with broadening of the internal diameter (2.02 cm); **c** and **d** on January 15, 2018 (34 weeks of gestation), fetal brain magnetic resonance imaging reveals abnormal morphology of the left frontal lobe with irregular extension of anterior horn of the left ventricle

**Fig. 4:**
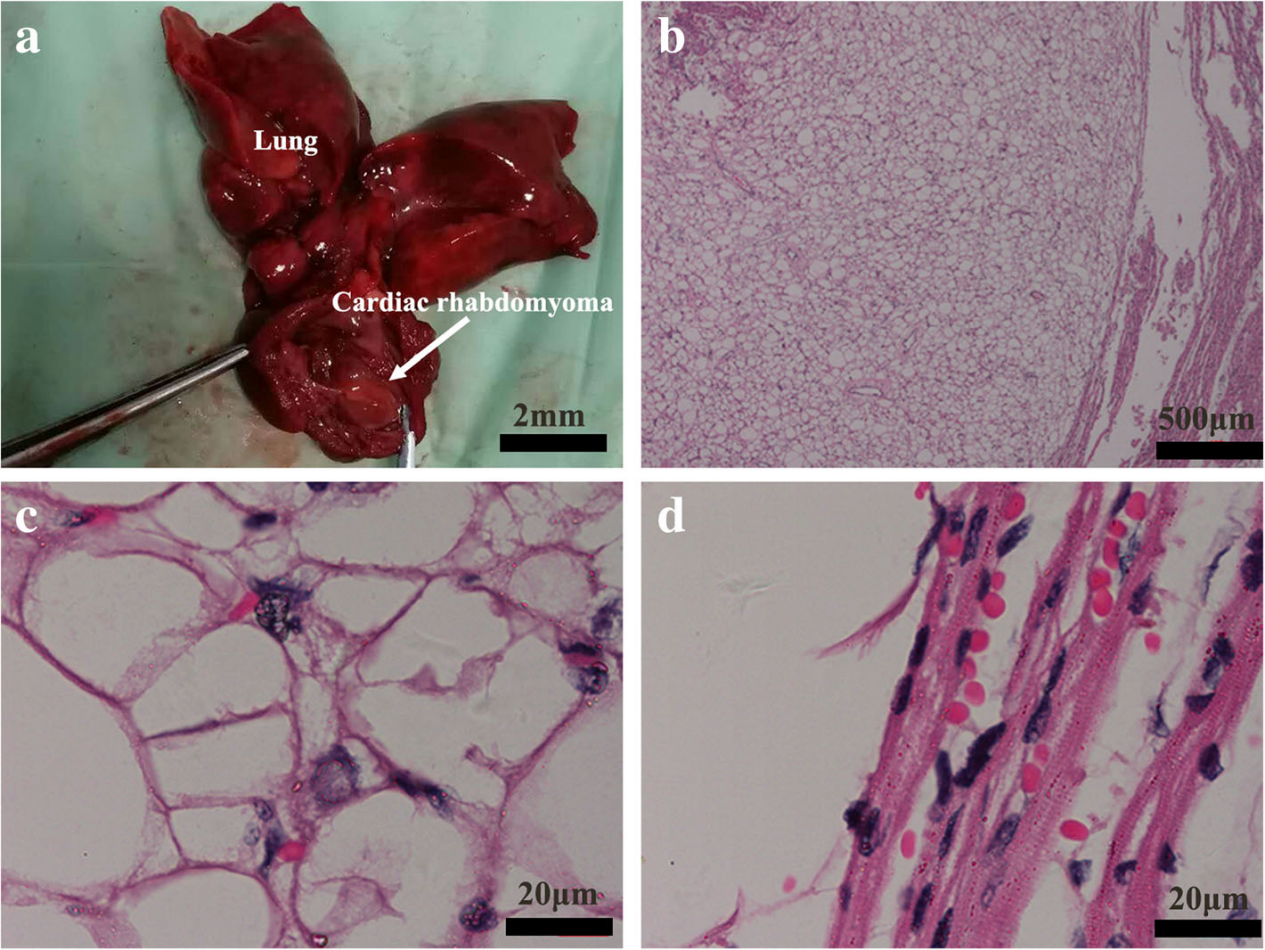
Anatomical and histological findings. **a** anatomical findings show a solid space-occupying lesion in the fetal left ventricle (approximately 4 cm × 3 cm); **b**, **c** and **d** histology of the fetal heart specimen reveals typical spider-shaped cells, which are consisted of abundant glycogen-rich cytoplasm and cytofilaments that are extended and radiated to pericellular regions, and pathologic examinations confirm that the solid space-occupying lesion in the fetal left ventricle is cardiac rhabdomyoma

At a gestational age of 28 + 3 weeks (December 7, 2017), color Doppler ultrasound reexaminations displayed multiple strong echoes in the fetal left ventricle (measuring 3.3 cm × 2.03 cm), compression of the left ventricular outflow tract, obvious enlargement of the tumor, and a 0.92 cm internal diameter of posterior horn of the left lateral ventricle (Fig. 1b). On January 15, 2018 (34 weeks of gestation), fetal brain magnetic resonance imaging (MRI) in Fujian Provincial Maternity and Children’s Hospital (Fuzhou, China) revealed abnormal morphology of the left frontal lobe with irregular extension of anterior horn of the left ventricle (Fig. 3c and d). Lissencephaly in the left frontal lobe and cortical dysplasia were therefore diagnosed. On January 25, 2018 (35 + 2 weeks of gestation), color Doppler ultrasound reexaminations of the fetal heart displayed multiple hyperechogenic masses (measuring 4.35 cm × 3.13 cm) in the fetal left ventricle, abnormal morphology of anterior horn of the left lateral ventricle with broadening of the internal diameter (2.02 cm) (Fig. 3a and b), indicating a high likelihood of cardiac rhabdomyoma. After careful consideration, the pregnant woman and her family decided to terminate pregnancy. Labor was induced and a 3 kg male infant was delivered. Anatomical findings showed a solid space-occupying lesion in the fetal left ventricle (approximately 4 cm × 3 cm) (Fig. 4a). Histology of the fetal heart specimen revealed typical spider-shaped cells, which were consisted of abundant glycogen-rich cytoplasm and cytofilaments that were extended and radiated to pericellular regions, and pathologic examinations confirmed that the solid space-occupying lesion in the fetal left ventricle was cardiac rhabdomyoma (Fig. 4b, c and d). Next-generation sequencing revealed no mutations in the tuberous sclerosis complex 1 (*TSC1*) gene or *TSC2* gene in the fetus. On August 12, 2018, peripheral blood was sampled from the fetus’ parents, and subjected to CMA. Then, a 2.5 Mb-duplication of the chromosome 15q13.2q13.3 region (arr [hg19] 15q13.2q13.3(30,386,398-32,915,089)× 3) was detected in the woman, and no mutations were identified in the TSC1 or TSC2 gene. These testing suggested that the fetal chromosome with the microduplication was inherited from his mother.

The pregnant woman reported a regular menstrual cycle, a natural pregnancy, and smooth pregnancy, and she had no special discomfort, no smoking or alcohol consumption history, no family history of fetal anomaly, and no history of viral infections. In addition, she denied oral administration of special medications during the pregnancy. During the pregnancy, non-invasive prenatal testing (NIPT) revealed a low risk, and color Doppler ultrasound imaging of the woman heart displayed no remarkable abnormality of cardiac structure or functions.

This study was approved by the Ethics Review Committee of Fuzhou Municipal First Hospital Affiliated to Fujian Medical University (approval no. FZSY-201700132). Written informed consent was obtained from the fetus’s parents following a detailed description of the study purpose. All experimental procedures described in this study were in accordance with international and national laws, regulations and guidelines. The pregnant woman and her husband involved in this study agreed to publish related demographic and clinical features.

## Discussion

CMA, also known as array comparative genomic hybridization or comparative genomic hybridization, is a high-resolutiongenome-wide screening testing to detect a majority of chromosomal imbalance and CNV (microdeletion and microduplication) [16–18]. As compared to the conventional karyotype analysis, CMA presents a higher resolution, which may detect 50 to 100 bp microdeletions and microduplications, and the CNV neighboring the rearranged breakpoints that were seemingly balanced in karyotyping [19–21]. It is therefore considered that CMA is replacing karyotyping as a first-line test in the evaluation of pregnancies in the era of omics [22–24].

In this study, routine color Doppler ultrasound imaging displayed fetal cardiac developmental defects, and further chromosomal G-banding revealed no abnormal karyotype; however, CMA detected a 1.8 Mb-duplication of the chromosome 15q13.2q13.3 region containing 7 genes (*TRPM1*, *KLF13*, *OTUD7A*, *CHRNA7*, *FAN1*, *MIR211* and *RAHGAP11A*), which occurred in the region between BP4-BP5 on chromosome 15q13.3. Neuronal acetylcholine receptor subunit alpha-7, also known as nAChRα7, a protein that in humans is encoded by the *CHRNA7* gene that contains 479 amino acids, is a major component of the neuronal nicotinic receptor, which is widely distributed in brain and spinal cord [25]. It has been reported that subjects with chromosome 15q13 microduplications may develop cognitive impairment, autism, seizures, and attention-deficit hyperactivity disorder, and oral clefts [6–14], and these disorders may be strongly associated with 15q13.3 microduplications involving *CHRNA7* [7, 9, 10, 14, 26, 27]. Scan statistic-based analysis of exome sequencing data showed that *FAN1* is a key driver in the 15q13.3 locus for the associated psychiatric and neurodevelopmental phenotypes, and *FAN1* at 15q13.3 has been identified as a susceptibility gene for schizophrenia and autism [28], while *MIR211* was identified as a putative attenuator of cholinergic-mediated seizures by intersecting forebrain microRNA profiles and dynamic miR-211 decreases were found to induce hypersynchronization and nonconvulsive and convulsive seizures, accompanied by expression changes in cholinergic and TGFBR2 pathways [29]. In addition, the *OTUD7A* gene has been identified as a critical gene regulating neurodevelopmental phenotypes [30], and the *TRPM1* gene is closely associated with fetal intelligence development [31–33]. In this study, the fetus harboring the 1.8 Mb-duplication of the chromosome 15q13.2q13.3 region containing the *CHRNA7*, *TRPM1* and *OTUD7A* genes, suggesting that a high possibility of developmental neuropsychiatric disorders in the fetus.

In a previous report, a recurrent duplication 15q13.3 (BP4-BP5) was detected in 150 patients with congenital heart defects, suggesting the possible link between chromosome 15q13 duplication and heart disorders [15]; however, the association between chromosome 15q13 microduplication and cardiac developmental defects has not fully undefined until now. In this study, the 1.8 Mb-duplication of the chromosome 15q13.2q13.3 region contained the *KLF13* gene. *KLF13*, an evolutionally highly-conserved gene, is a member of the Krüppel-like family that encodes zinc-finger proteins, which mainly functions as a regulator of cardiac gene expression and heart development by interacting with GATA4 [34, 35]. Immunohistochemical staining showed high KLF13 protein expression in myocardial and endocardial tissues [35]. In addition, knockdown of *KLF13* in Xenopus embryos was found to cause abnormal cardiac development, including ventricular trabecular formation, atrioventricular septal defects, delayed atrioventricular cushion formation and delayed heart valve maturation [36]. In this study, cardiac ultrasound follow-up revealed significant enlargement of the space-occupying lesion in the fetal heart with the extension of the gestational age, and the space-occupying lesion was finally pathologically diagnosed as cardiac rhabdomyoma. In addition, no mutations were identified in the *TSC1* or *TSC2* gene in the fetus, the mother or the father by next-generation sequencing, which may rule out tuberous sclerosis complex [37, 38]. Taken these findings together, it is hypothesized that the development of fetal cardiac rhabdomyoma may be attributed to the aberrant *KLF13* expression caused by chromosome 15q13 microduplication, which is in agreement with previous studies [15]; however, more clinical evidence is required to examine the association between chromosome 15q13 microduplication and cardiac rhabdomyoma.

## Conclusions

The results of the present study demonstrate, for the first time, the potential association between chromosome 15q13 microduplication and fetal cardiac rhabdomyoma. Since CMA is effective to detect hereditary diseases that G-banding karyotyping fails to do [19–21], it is therefore recommended that CMA be employed in fetuses with abnormal cardiac development diagnosed by routine cardiac color Doppler ultrasound imaging for early detection of congenital genetic abnormality, which may provide valuable information for prenatal diagnostic consultation and the decision on pregnancy termination.

## Data Availability

The data reported in this study are available upon request by contact with the corresponding author.

## Abbreviations

CMA: Chromosomal microarray analysis
CNV: Copy number variation
NIPT: Non-invasive prenatal testing
